# A multi-organ-on-chip to recapitulate the infiltration and the cytotoxic activity of circulating NK cells in 3D matrix-based tumor model

**DOI:** 10.3389/fbioe.2022.945149

**Published:** 2022-07-25

**Authors:** Monica Marzagalli, Giorgia Pelizzoni, Arianna Fedi, Chiara Vitale, Fabrizio Fontana, Silvia Bruno, Alessandro Poggi, Alessandra Dondero, Maurizio Aiello, Roberta Castriconi, Cristina Bottino, Silvia Scaglione

**Affiliations:** ^1^ React4life S.r.l, Genoa, Italy; ^2^ Department of Biotechnology and Bioscience, University of Milano-Bicocca, Piazza Della Scienza, Milan, Italy; ^3^ National Research Council, CNR-IEIIT, Genoa, Italy; ^4^ Department of Experimental Medicine (DIMES), University of Genoa, Genoa, Italy; ^5^ Department of Pharmacological and Biomolecular Sciences (DiSFeB), University of Milan, Milan, Italy; ^6^ Molecular Oncology and Angiogenesis Unit, IRCCS Ospedale Policlinico San Martino, Genoa, Italy; ^7^ IRCCS Istituto Giannina Gaslini, Genoa, Italy

**Keywords:** immune-organ-on-chip, 3D human tumor model, natural killer cells, neuroblastoma, cells migration, cells infiltration

## Abstract

The success of immunotherapeutic approaches strictly depends on the immune cells interaction with cancer cells. While conventional *in vitro* cell cultures under-represent the complexity and dynamic crosstalk of the tumor microenvironment, animal models do not allow deciphering the anti-tumor activity of the human immune system. Therefore, the development of reliable and predictive preclinical models has become crucial for the screening of immune-therapeutic approaches. We here present an organ-on-chip organ on chips (OOC)-based approach for recapitulating the immune cell Natural Killer (NK) migration under physiological fluid flow, infiltration within a 3D tumor matrix, and activation against neuroblastoma cancer cells in a humanized, fluid-dynamic environment. Circulating NK cells actively initiate a spontaneous “extravasation” process toward the physically separated tumor niche, retaining their ability to interact with matrix-embedded tumor cells, and to display a cytotoxic effect (tumor cell apoptosis). Since NK cells infiltration and phenotype is correlated with prognosis and response to immunotherapy, their phenotype is also investigated: most importantly, a clear decrease in CD16-positive NK cells within the migrated and infiltrated population is observed. The proposed immune-tumor OOC-based model represents a promising approach for faithfully recapitulating the human pathology and efficiently employing the immunotherapies testing, eventually in a personalized perspective. An immune-organ on chip to recapitulate the tumor-mediated infiltration of circulating immune cells within 3D tumor model.

## Introduction

Immunotherapies represent one of the current most promising challenges in cancer treatment. These are based on the concept of boosting the host’s immune system toward the elimination of cancer and include several strategies such as the use of monoclonal antibodies (mAb) targeting tumor-associated molecules or blocking immune checkpoints, anti-cancer vaccines and cell-based therapies (Ringquist et al., 2021; Shelton et al., 2021). The success of immunotherapeutic approaches aimed to unleash the activity of cytotoxic cells such as CD8^+^ T lymphocytes or Natural Killer (NK) cells strictly depends on their interaction with cancer cells and other immune cells. Such interaction might be profoundly affected by the highly complex niche of the tumor microenvironment (TME), which is populated by different type of cells (i.e., stromal cells, tumor cells, immune cells), interconnected within a complex three-dimensional vascularized matrix. Given these premises, the development of reliable preclinical human models has become crucial for the assessment of the best immune therapeutic approaches (Boucherit et al., 2020; Ando et al., 2021; Ringquist et al., 2021).

To date, preclinical safety and efficacy assessment of immunotherapies are carried out through 1) extensive *in vitro* cultures, addressing the cellular and molecular basis of immune responses, cancer initiation and development, and its interactions with immune cells 2) *in vivo* xenografts and genetically engineered animal models, for a necessary systemic contextualization. However, both approaches show limitations. Conventional *in vitro* models use 2D cultures that under-represent the complexity of the TME (three-dimensionality, shear stresses due to fluidic exposure, irroration affecting molecule distribution) (Rodrigues et al., 2021). On the other side, the animal models classically used in oncology often rely on immunodeficient mice xenografted with human cell lines or patient-derived tumor cells (PDX). These models, although useful for testing anti-tumor drugs, do not allow to obtain information regarding the anti-tumor activity of the human immune system. Other *in vivo* approaches are based on syngeneic mice with a fully competent immune system with results that not always recapitulate the human setting (Franklin et al., 2022). The more sophisticated and informative animal models to date are the so called “humanized mice” having a human HSC-derived immune system which can be engrafted with human tumors. However, some limitations are related to the often incomplete reconstitution of the human immune system, in terms of cell lineage development, wrong proportion of the various immune cell subtypes and degree of their activity and maturation. These defects are due to the presence of a mouse-specific microenvironment lacking human stroma and growth factors (Cogels et al., 2021). Moreover, such models are very expensive, time-consuming, and hardly usable in personalized medicine.

Therefore, 3D, human *in vitro* tumor models, including spheroids and organoids, as well as microfluidics approaches integrating the human immune components, are increasingly being developed and adopted (Rodrigues et al., 2021; Vitale et al., 2022). 3D tumor models have advantages over conventional 2D cultures, such as an increased cellular and architectural cancer complexity, like the presence of a biomimetic TME with the proper chemical and biomechanical features (Cavo et al., 2016), beside to the possibility of coculturing stromal, endothelial and cancer cells where cells can migrate and infiltrate in a 3D space. Moreover, from a technical point of view, the adoption of 3D cancer models allows to scale up cell yields, with a sample size compatible with a wide variety of standard downstream analysis, such as molecular and cytofluorimetric analysis, beside immunohistochemistry maintaining a possible anisotropic cells behavior (Ballester-Beltran et al., 2015).

The arising microfluidic platforms display the potential to recapitulate the physiological blood flows affecting the survival of circulating tumor cells (Marrella et al., 2021a), the intra/extravasation of circulating cells (tumor cells or activated immune cells), besides a reliable drug distribution (Marrella et al., 2021b). However, some microfluidic models used to co-culture immune and tumor cells in physically separated compartments are currently adopted in static conditions (Lee et al., 2018). Moreover, the over-miniaturization of some organ on chips **(**OOC) allow to host only few cells and very small amount of tumor samples, thus limiting the downstream analytical approaches.

From a manufacturing point of view, the conventional OOC models and microfluidic models under development are typically fabricated using the polydimethylsiloxane (PDMS) elastomer, in which UV lithography is utilized to create an overall chip architecture with microscale fluid channels across the compartments where few microliters of media circulate without any sampling/injection port (Amin et al., 2016; Lee et al., 2018). Besides suffering the difficulty of mimicking the complex structures of the microenvironment *in vivo* (Amin et al., 2016), these PDMS-based devices lead to the adsorption of small hydrophobic compounds on the chip, causing the reduction of their bioavailability, finally resulting in issues in terms of cellular responses and/or *bias* in biochemical analysis.

In this paper, we present a unique and promising approach aimed at recapitulating the immune cell infiltration and activation against cancer cells in a humanized, fluid-dynamic and 3D environment. An organ-on-chip technology (MIVO^®^) has been recently adopted by authors for recapitulating the systemic administration of anticancer drugs and for carrying out efficacy assays in comparison with the standard xenograft model, demonstrating the high predictability of this *in vitro* 3R approach (Marrella et al., 2021b). Similarly, the same technological platform has been adopted for culturing aggressive breast cancer models and recapitulating the cancer cells migration and infiltration in the fluid flow circuit, which represents the first physio-pathological step towards the metastatic onset (Cavo et al., 2018). Here, the generation of a humanized and immunocompetent *in vitro* cancer model (Marrella et al., 2019), where tumor cells are cultured in a compartment physically separated through a porous permeable membrane from the fluid flow compartment, relies on the capability to emulate the microcirculation as well as the circulatory behavior of immune cells within the TME. The possible access to both the tumor and the circulating compartments allows to monitor and quantify the changes that occur in the TME (soluble molecules, cell death, tumor cell invasion), in circulating immune cells, and potentially in additional compartments physically connected each other through the circulating fluid flows (i.e., evaluation of the metastatic site). The flexibility of this approach carries the important potential of better recapitulating a clinical scenario, opening the way for a more reliable platform for 1) a personalized investigation of the specific migratory and infiltrative capacity of immune cells, 2) the analysis of the anti-tumor activity of both drug-based and cell-based therapies, 3) the investigation of the effects of tumor-immune cell cross-talk often leading to the onset of resistant tumor variants (Bottino et al., 2021).

## Results

### Assessment of natural killer cell viability in standard 2D culture vs*.* 3D alginate embedding

High-risk neuroblastoma (NB) is an aggressive, metastatic pediatric cancer difficult to treat and still characterized by poor overall survival. We have recently developed an alginate-based 3D NB culture as *in vitro* model characterized by a more physio-pathological setting showing only a partial overlap with the standard 2D culture in terms of expression of immune-related molecules (Marrella et al., 2019). We also demonstrated that the NB cell viability and proliferation are preserved after embedding NB cells within the 3D alginate matrix.

The aim of the present work is the implementation of such three-dimensional culture with features mimicking 1) the dynamic microcirculation within the tumor microenvironment and 2) the circulatory behavior of immune cells. Then, a reliable dynamic culture, consisting in the 3D NB model cultured with circulating NK cells, has been generated as a suitable tool for analyzing immune cell migration and infiltration, as well as tumor cell killing.

Given the hypothesis that circulating NK cells could be able to specifically infiltrate the alginate-based NB culture, we wanted to verify whether the alginate matrix per se could be detrimental for the NK cell viability. NK cells were cultured in standard conditions (suspended culture within a 96-well plate) or embedded in the alginate-based 3D matrix; the cells were then collected from the standard culture by pipetting or recovered from the 3D culture through alginate dissolution, stained with a Viability Dye, and analyzed by flow cytometry.

**FIGURE 1:**
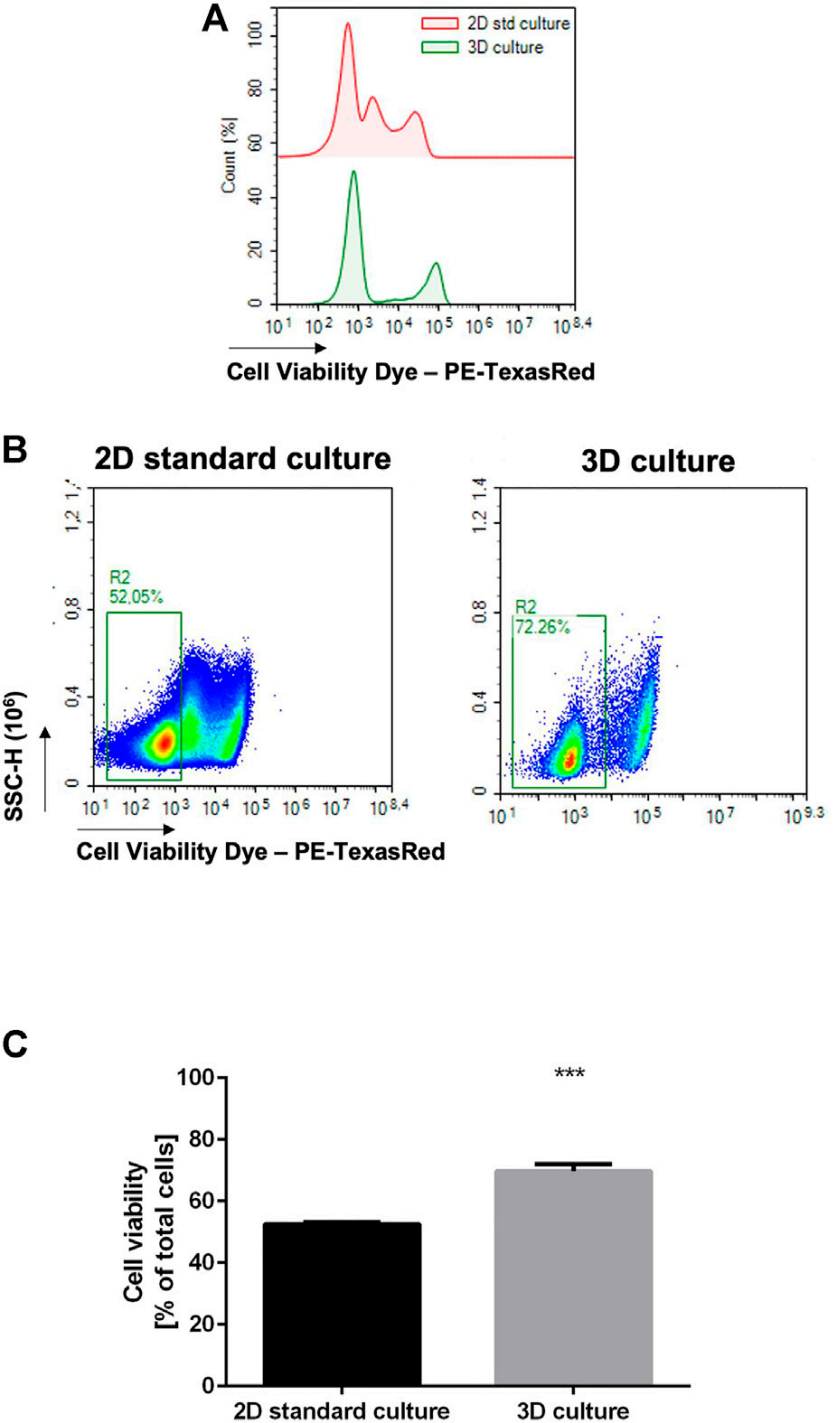
3D alginate embedding does not negatively affect cell viability. NK cells were cultured in the standard setting (*n* = 6) or in 3D alginate hydrogel (*n* = 6) for 24 h. The cells were then recovered and stained with a Fixable Cell Viability Dye (PE-TexasRed). **(A)** Flow cytometry analysis on NK cells, histograms: three distinct populations are observed in the 2D standard culture. **(B)** Flow cytometry analysis on NK cells showing % of low-staining population. **(C)** Statistical analysis of **(B)**.

In the NK 3D culture, a clear population of unstained (thus viable) cells is shown beside to a clear stained population of dead cells (Figure 1A, lower histogram); conversely, in the 2D culture the presence of a mid-stained population might be indicative of early cell death/structural degradation of the cell, enriching the population of non-viable cells (Figure 1A, upper histogram). According to the clearly viable population, a significant difference was observed in cell viability between the standard suspended NK culture and the 3D embedding (Figures 1B,C).

### Reliability of flow cytometry analysis after cell recovery

**FIGURE 2:**
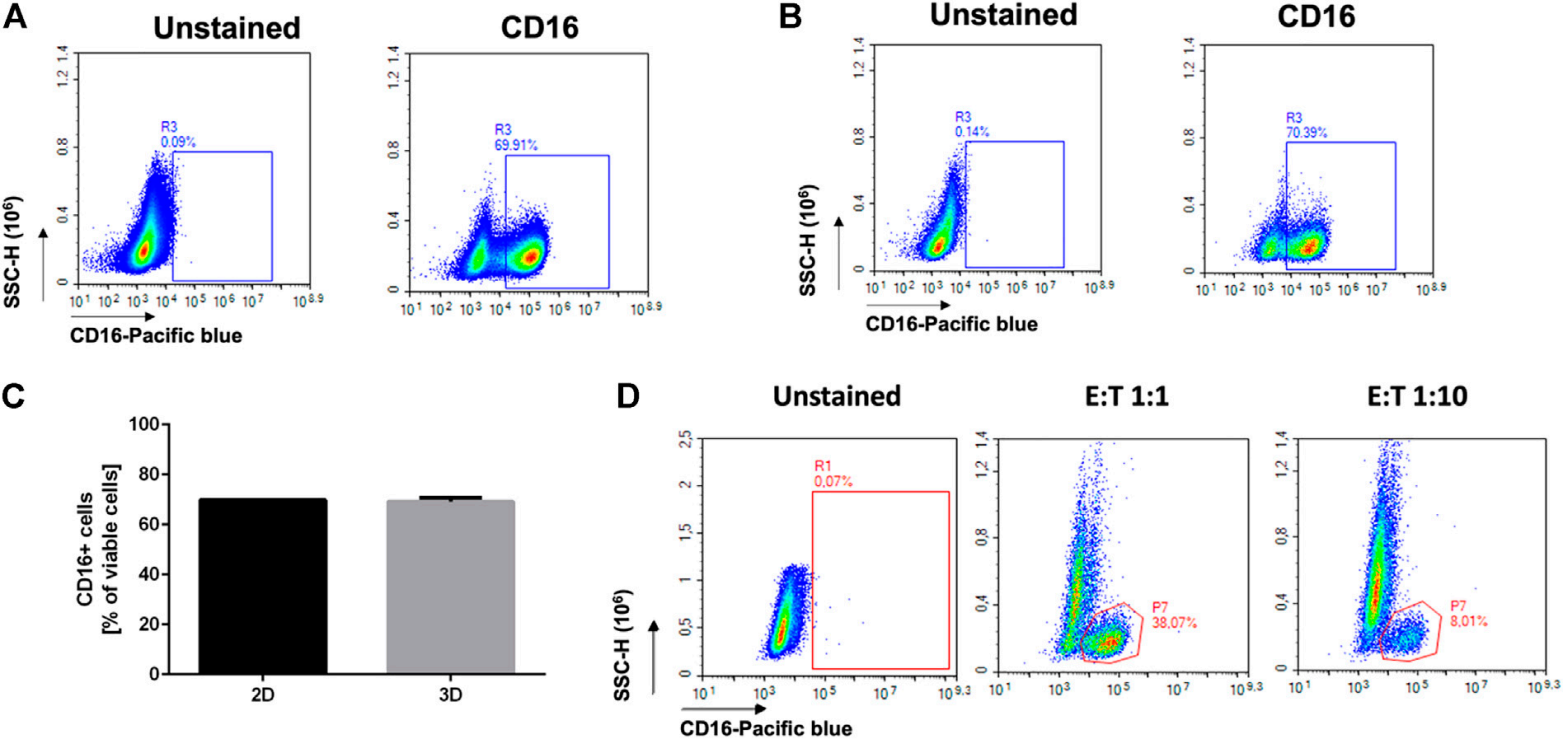
Reliability of flow cytometry analysis after cell recovery. NK cells were cultured in the standard 2D setting **(A)**, in 3D alginate embedding **(B)**, or co-cultured with tumor cells in the 3D alginate matrix **(C)**. The cells were then recovered and analyzed for the expression of the NK marker CD16. **(A)** CD16 expression on NK cells cultured in standard conditions (96-well plate, n = =6). **(B)** CD16 expression on NK cells cultured in a 3D alginate scaffold (n = =6). **(C)** No significant difference in CD16 expression is highlighted by flow cytometry in standard vs. 3D culture. **(D)** CD16 expression on NK cells recovered from a 3D alginate-based co-culture with HTLA-230 cells, at different E:T ratios (n = =12).

Flow cytometry is one of the most widely adopted approaches for quantitative and qualitative assessment of the composition of TME. With the aim to generate a reliable *in vitro* model, to support some preclinical studies in a 3Rs perspective, we wanted to check if flow cytometry could be successfully employed to analyze heterogeneous cell populations recovered from the alginate scaffold, as a model of a complex 3D tumor. We started by analyzing simple NK monocultures. NK cells were cultured in standard 2D conditions or in 3D embedding over/night, and the cells directly collecting from the cell suspension or dissociating the alginate, respectively. We assessed the expression of the NK-associated marker CD16, being aware that such marker is highly expressed in polyclonal NK cells (Figure 2). Indeed, as expected, we found high percentage (70%) of CD16^+^ positive cells (Figures 2A,B). No significant differences were observed between the 2D and 3D culture setting in terms of percentage (Figure 2C) of positive cells (69.91% vs. 70.39%).

We then prepared cocultures with NK and the HTLA-230 NB cell line, to assess the reliability of the staining of hetero-cultures. In particular, we performed cocultures with two different effector/target (E: T) ratio (1:1 and 1:10)*.* The lowest NK: NB ratio was specifically chosen with the aim to get closer to *in vivo* scenario where few NK cells generally infiltrate the most aggressive solid tumors including NB (Balsamo et al., 2012; Castriconi et al., 2018; Melaiu et al., 2020).

We then verified by flow cytometry the percentage of CD16-positive cells, gated on total cells. Considering that, in the NK monocultures, approximately 70% of the cells show CD16 positivity (Figures 2A–C), as expected, we detected around 38% CD16^+^ cells in the 1:1 coculture and around 8% CD16^+^ cells in the 1:10 coculture.

### Assessment of the natural killer-tumor cell interaction within the 3D model

To verify if NK cells could infiltrate the alginate matrix and interact with tumor cells, we co-cultured NK cells and NB at different E:T ratios (1:1 and 1:10).

**FIGURE 3:**
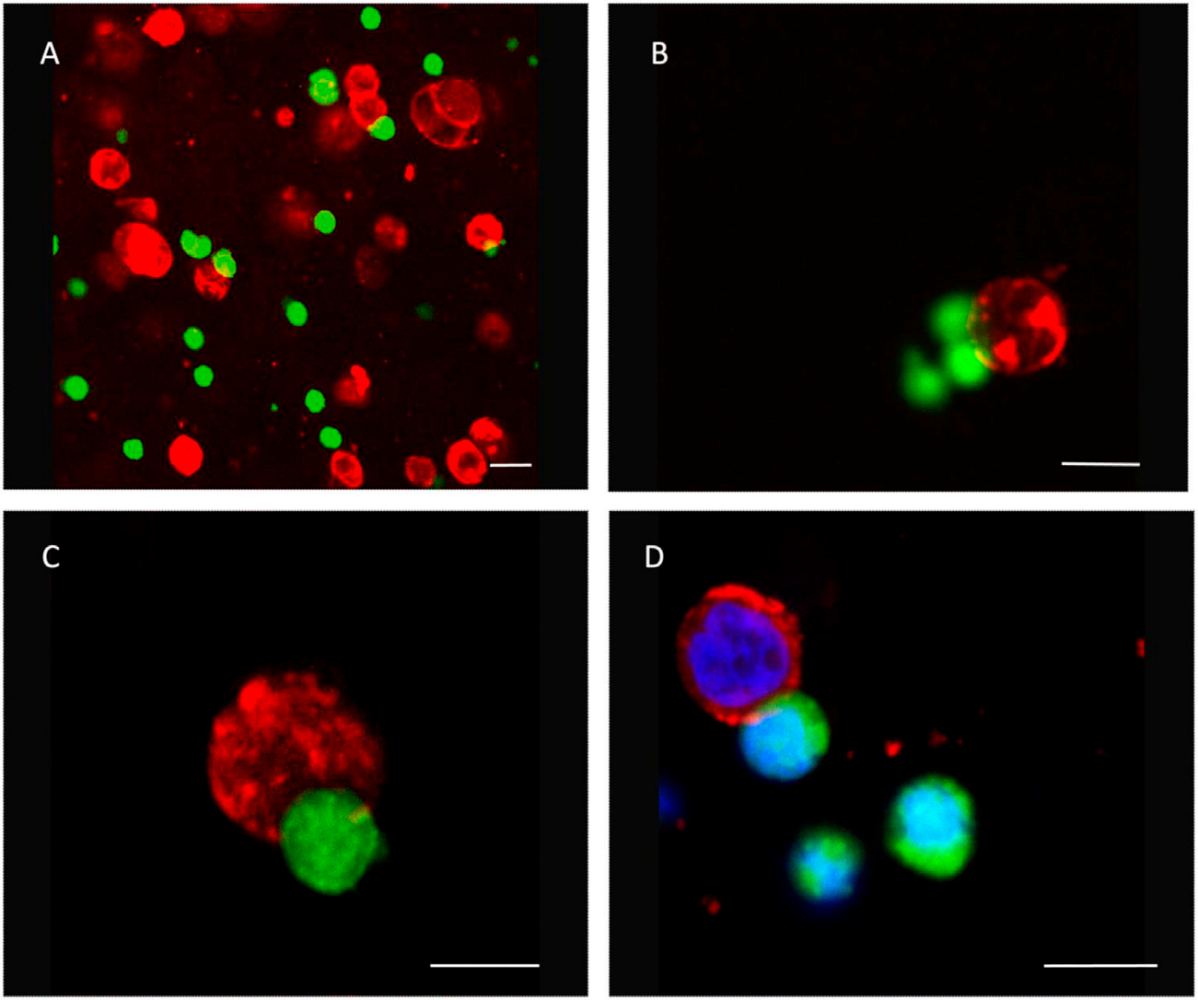
NK cells and NB cells coembedded in a 3D alginate scaffold. Confocal Microscopy Acquisition and Analysis of CFSE-labeled NK cells (green) and GD2-positive NB cells (red) co-embedded in 3D alginate scaffold and cultured for 24 h. In panel D, nuclei have been stained with DAPI. **(A)** ×20 magnification (representative square field of ×173173 micron); **(B–D)** ×40 magnification. Scale bar: 10 micron.

When embedded in the alginate matrix, NK cells and tumor cells are homogeneously dispersed in a single-cell suspension. To assess the ability of NK cells to move within the matrix for reaching the tumor cells, we performed a confocal microscopy analysis. NK cells were labelled with the cell tracker CFSE (green) and then co-embedded with the unlabeled tumor cells. Then, the 3D co-culture was fixed in paraformaldehyde and stained with a mAb specific for the NB-specific marker GD2 (red). We observed several NK cells start to interact with NB cells (Figure 3).

**FIGURE 4:**
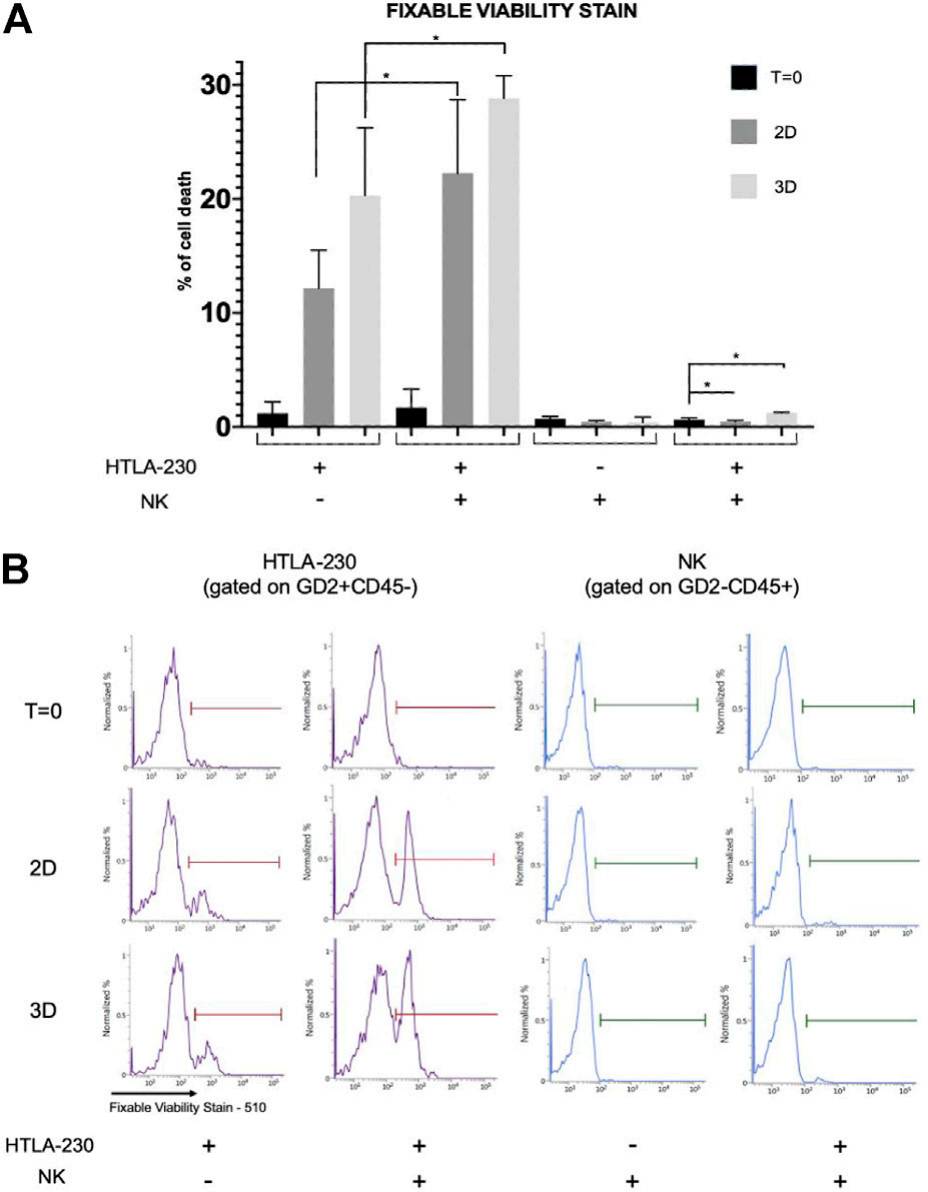
Viability assessment of NB and NK cells in 2D and 3D cultures. **(A)** Percentage of dead NB or NK cells (i.e., Fixable Viability Stain 510 positive cells) recovered from 2D cultures or 3D alginate spheres, both in monoculture and coculture, gated on GD2+CD45− and CD45+GD2−, respectively. Mean fluorescence intensity with 95% CI is reported (*n* = 4, *p < 0.05). **(B)** Representative experiment.

This leads to a significant tumor cell death paralleled by a high NK cell viability (Figure 4): cells co-culture analysis showed that tumor cell death significantly increased when NB cells were co-cultured with NK cells in 3D models, indicating that NK cells are able to interact and effectively kill tumor cells grown in 3D cultures. As expected, the percentage of dead tumor cells in the presence of NK cells was also significantly increased in 2D culture, whereas at T = 0 was negligible. Moreover, NK cells recovered from hydrogels showed a high viability, demonstrating that 3D alginate spheres are suitable models for testing NK cell-mediated immunotherapy.

### Dynamic culture and assessment of natural killer cell “extravasation”

**FIGURE 5:**
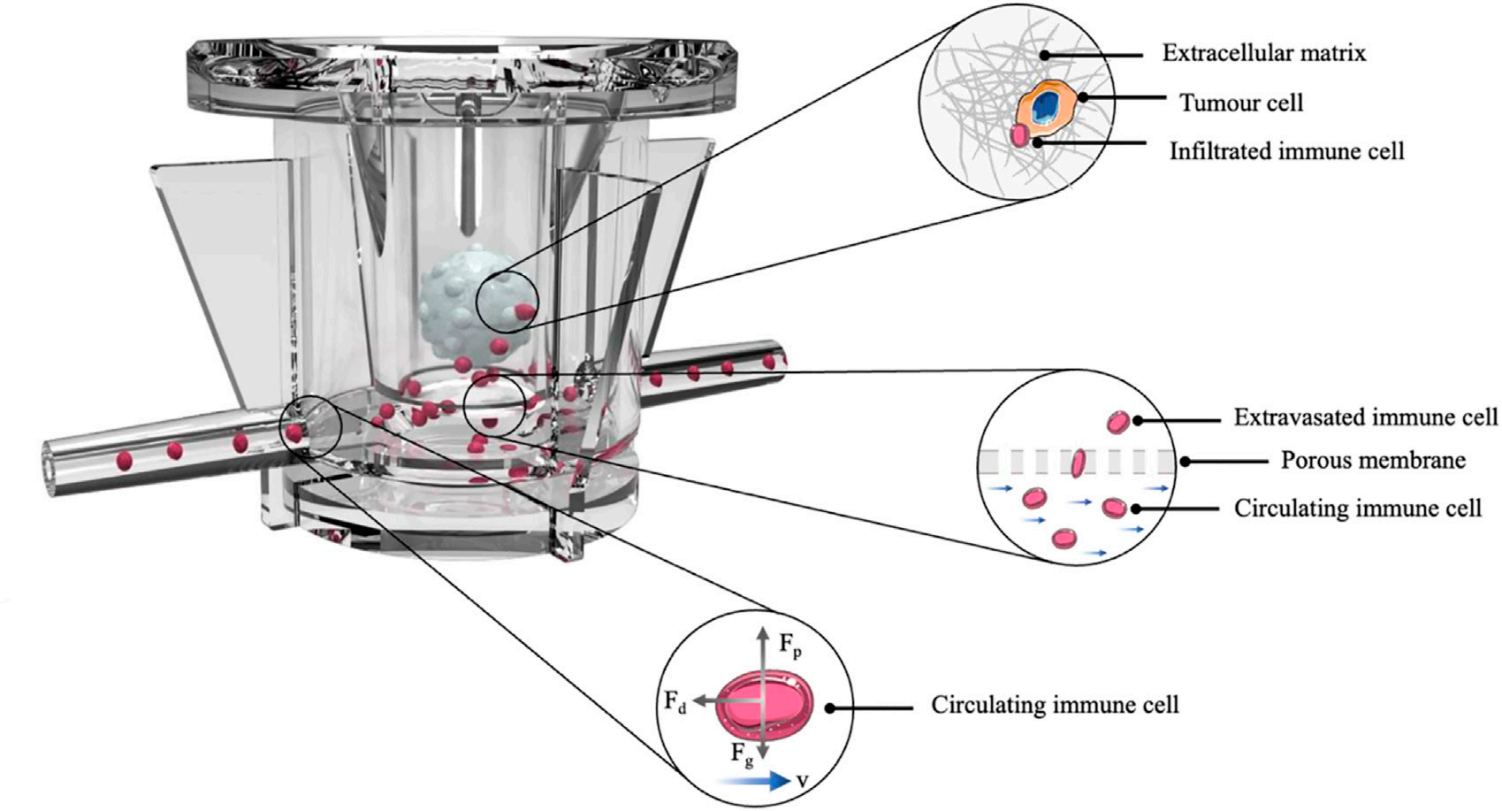
Organ on chip platform for immune-tumor cells cross-talk. Representative scheme of the experiment: HTLA-230 cells were embedded in a 3D alginate scaffold and cultured above a microcirculation of NK cells, for 4 h.

Once we have analyzed the feasibility of 3D cultures in static conditions, we carried out the dynamic co-culture by using MIVO^®^. The dynamic culture has been specifically planned to allow the circulation of NK cells below hydrogel-embedded tumor cells (E:T ratio 10:1), which are accommodated in a standard 24-well plate transwell insert, fitting the MIVO^®^ chamber (Figure 5).

**FIGURE 6:**
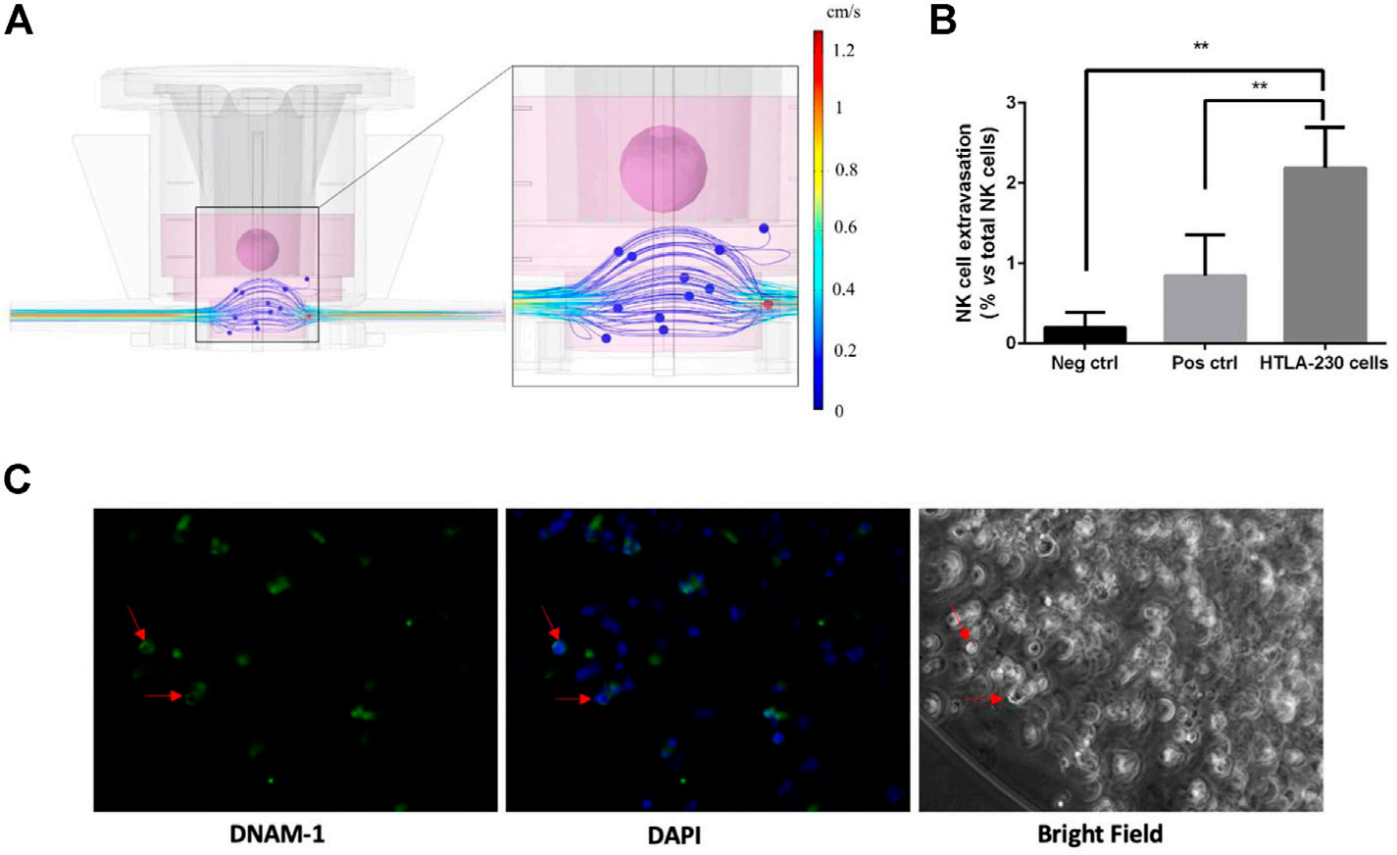
Dynamic culture with circulating NK cells (4 h coculture). **(A)** CFD simulation of the fluid velocity profiles within the organ on chip and the fluid flow-driven immune cells trajectories. **(B)** Tumor-specific NK cell extravasation (neg ctrl: DMEM w/o supplements; pos ctrl: alginate gels w/o HTLA-230 cells, plus 30% FBS; HTLA-230 gels with standard 10% FBS) (*n* = 6). **(C)** NK cell infiltration within the gel, as indicated by DNAM-1-positive cells highlighted by red arrows.

The device has been connected to a pumping system, which enable the imposition of a fluid flow, driving NK cells throughout the circulation, thus mimicking the circulatory system within the tumor microenvironment. The MIVO material is PDMS free, to avoid any molecule binding issues, and no immune cells adhesion at the walls was observed (data not shown). As evidence of that, Computational Fluid Dynamics (CFD) simulations have been performed. Results show that velocity values within MIVO^®^ range from 0 to 1.2 cm/s, which are characteristic of capillary blood flow, when the imposed inlet flow rate is 0.3 ml/min (Figure 6A).

We then verified the ability of NK cells to migrate upward (thus against the gravity force), following an active, chemoattractant-driven “extravasation”, with no driving forces dependent on the fluid flow per se. To this purpose, additional experimental groups included 1) a positive control of cell migration (“empty” 3D alginate scaffolds, without tumor cells, in culture medium enriched with 30% FBS as a chemoattractant), and 2) a negative control (without alginate scaffolds, with culture medium without any chemoattracting supplement). The tumor samples were represented by 3D alginate cultures of HTLA-230 cells, kept in standard conditions (cell culture medium with 10% FBS). After co-culturing the cells for 4 h in a dynamic microenvironment, the supernatants into the transwell inserts were collected, and the migrated cells were counted by means of a hemocytometer. As shown in Figure 6B, we did observe a significant increase of cell migration in the tumor group as well as in the positive control, whereas little or no NK cell migration was observed in the negative control group, demonstrating an active, tumor-specific, NK cell “extravasation”. The biochemical-driven specificity of the “extravasation” process is further corroborated by the CFD simulation aimed at investigating fluid flow-driven NK cells trajectories: the simulation does not report cells “extravasation” events from the bottom chamber towards the tumor, because of the fluid motion alone, as shown in Figure 6A.

### Natural killer cell infiltration

To understand if the migrated NK cells were capable to infiltrate the 3D tumor despite the presence of the alginate matrix, and eventually to interact with tumor cells, we performed the same dynamic culture described above. After co-culturing the cells for 4 h under microcirculation, the 3D alginate cultures were recovered, fixed with 4% PFA, NK cells stained for the DNAM-1 marker (Castriconi et al., 2004), and observed under a fluorescence microscopy. As shown in Figure 6C, DNAM-1+ cells were mostly located along the border of the gels. This is indicative of the ability of NK cells not only to specifically migrate toward the tumor culture, but also to infiltrate the alginate matrix.

**FIGURE 7:**
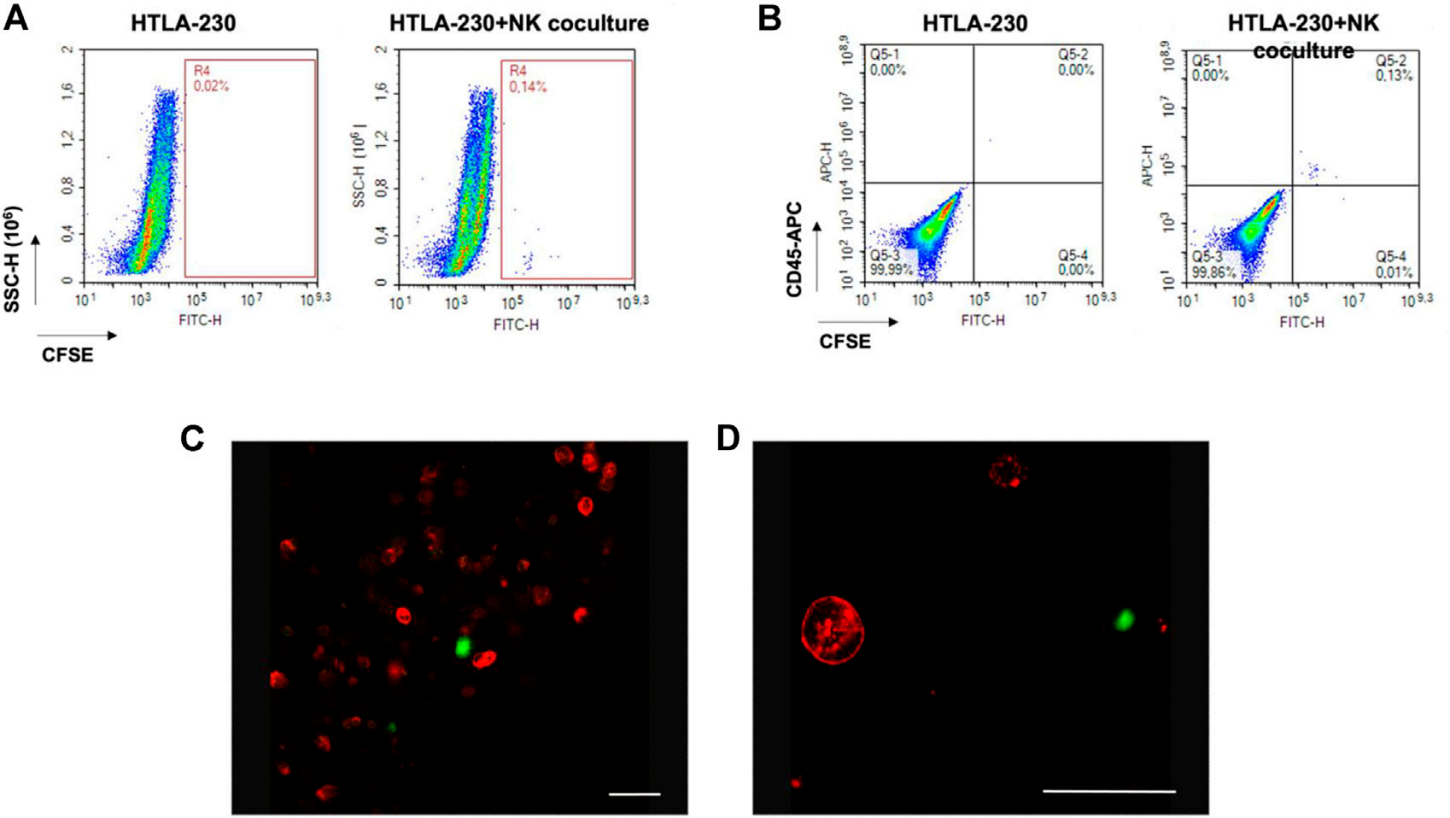
Over/night dynamic co-culture of alginate-embedded neuroblastoma cells with circulating NK cells. **(A)** Representative experiment showing a flow cytometry analysis of the cells recovered from the gels. A clear population of CFSE-labelled cells (corresponding to NK cells) is visible in the co-cultured sample (*n* = 6). **(B)** Perfect concordance between CFSE-labelling and CD45 expression (*n* = 6). **(C and D)** Confocal Analysis of different 3D alginate scaffold isolated from 3D dynamic cultures (4 h). Representative confocal microscopy xy fields (370 × 370 micron) acquired in 3D alginate scaffolds containing GD2-positive NB cells (red) that were previously embedded in the gel, and subjected to 4 h of dynamic flow of CFSE-labeled NK cells (green). Merged fluorescence images show that NK cells were able to infiltrate the gel. Scale bar is 50 micron.

The same experiment was performed by labelling NK cells with the cell tracker CFSE before the establishment of the coculture, and then following their journey toward the NB culture (Figures 7A,B). This served also as a proof of concept that all the cells found within the transwell inserts during the dynamic culture were CFSE-positive, thus NK only. No tumor cells migrated outside the alginate gels, excluding a bias in the quantification of the “extravasation” of immune cells (data not shown). After 4 h dynamic co-culture, the 3D alginate gels were recovered, fixed and stained for the tumor marker GD2, and observed under confocal microscopy, with the aim to highlight possible effector-to-tumor cell interactions. As shown in Figures 7C,D, CSFE-positive NK cells were found among the GD2-positive tumor ones within the alginate cultures.

### Assessment of the natural killer cell phenotype in circulating, extravasating, and infiltrating populations

The NK cell phenotype is critical for their anti-tumor effect, with CD16-positive NK cells being related to a high cytotoxic activity. However, because of the tumor microenvironment, the CD16-negative infiltrating NK cells may prevail, which are characterized by low-cytotoxicity.

Given these premises, we sought to verify if our dynamic culture could recapitulate the selection of a specific NK cell phenotype in the extravasated and infiltrated NK cell fraction. We then performed a dynamic culture, incubating HTLA-230 3D hydrogels with circulating, CFSE labelled NK cells over/night (10:1 E:T ratio). Then, we collected 1) the 3D tumor hydrogels, 2) the migrated NK cells, found into the transwell inserts, and 3) the circulating NK cells, which were analyzed for CD45 and CD16 expression through flow cytometry. The hydrogels were dissociated as described above, and the single cell suspension was analyzed identifying the CSFE-labelled NK cells infiltrating the tumor model. As shown in Figure 7A a clear, although small, CSFE-labeled cell population was present within the hydrogel, corroborating the findings (Figures 7C,D) on the ability of the “extravasated” NK cells to infiltrate the 3D tumor culture. As a further demonstration, CD45 staining confirmed that all and only the CFSE-positive cells were belonging to the immunity lineage (Figure 7B).

**FIGURE 8:**
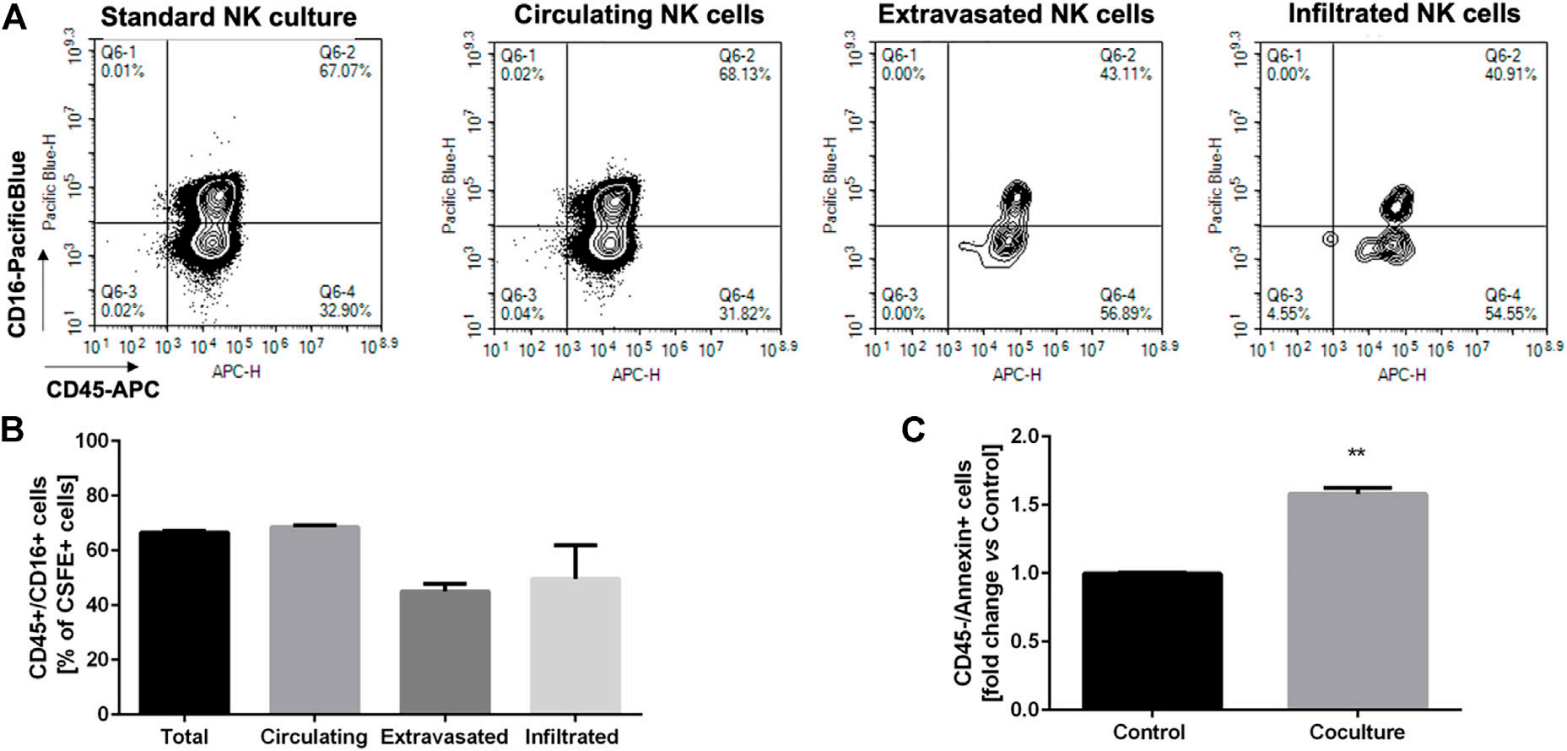
Phenotypic characterization of the different fractions of NK cells **(A)** Representative plotes showing the phenotypic characterization of the different fractions of NK cells recovered from the dynamic culture (infiltrated, extravasated, circulating vs*.* the standard NK culture) based on CD45/CD16 expression, gated on the CFSE-positive population (*n* = 6). **(B)** Statistical analysis of CD16 expression in the different fractions of NK cells, recovered from the dynamic culture (*n* = 6). **(C)** Induction of apoptosis in tumor cells, assessed through flow cytometry as annexin-V staining on CD45-negative cells (*n* = 6). Control: HTLA-230 3D monoculture.

Moreover, Figure 8 shows the proportion of CD45 and CD16 co-expressing NK cells, gated out on the total CFSE-positivity. We compared the standard NK culture (positive control), the circulating NK cells recovered from the MIVO^®^ devices, the migrated NK cells recovered from the transwell inserts, and the infiltrated NK cells recovered from the hydrogels. As highlighted in the representative plots in Figure 8A, as well as in Figure 8B, the proportion of NK cells expressing CD16 is markedly reduced in the “extravasated” and infiltrated groups, if compared with the circulating group, which conversely is similar to the standard NK culture. This might reflect a preferential recruitment of CD16-negative NK cell population, recapitulating data observed in different solid tumors (Balsamo et al., 2012; Castriconi et al., 2018; Melaiu et al., 2020).

Finally, we aimed at analyzing the induction of apoptosis in tumor cells, after overnight coculturing with circulating NK cells. Figure 8C shows a significant increase in the exposure of annexin-V on tumor cells membrane, indicating that the migrated infiltrating NK cells are able to specifically induce tumor cell death, thus retaining, at least in part, their cytotoxic potential.

## Discussion

The improvement of preclinical cancer models represents the basis for the acceleration of the development of more effective and personalized therapeutic strategies, and the reduction of clinical failure rate of oncology drugs, mainly due to the lack of both 1) complex clinically relevant *in vitro* models that better recapitulate the physio-pathological features occurring in patients (Jardim et al., 2017; Wong et al., 2019; Honkala et al., 2021), and 2) fully humanized animal models (Mak et al., 2014; Maulana et al., 2021; Bjornson-Hooper et al., 2022). For instance, and importantly, immune-oncology therapies would need a fully integrated human microenvironment to be tested, given the intricate interplay between the different immune cell subsets, tumor cells, and other cells within the TME. Moreover, the vascularization state, the accessibility of the tumor bulk and the presence of immunosuppressive signals within the human TME must be considered for both drug-based and cell-based immunotherapies.

Starting from a two-dimensional “flat biology”, across the development of three-dimensional cultures and co-cultures, the current rise of microfluidic technologies enables the implementation of a “fourth dimension”, with the introduction of fluid flows mimicking the tissue dynamic environment. In this context, the organ-on-chip technology represents a new generation of *in vitro* models, which consists in the realization of hyper organized cell cultures building tissue-level structures, the corresponding physiological functions (Bhatia and Ingber, 2014; Sontheimer-Phelps et al., 2019) and different cellular compartments which can cross-communicate through channels interconnection, and/or porous membranes (Maulana et al., 2021).

For instance, the cultivation of cancer cells and immune cells in two separated compartments, connected through microchannels within the same planar chip, allows the establishment of a biochemical gradient for immune cell recruitment from the first chamber toward the side chamber, hosting tumor cells. However, most of these tumor-on-chip are used either in static conditions (Hsu et al., 2012; Businaro et al., 2013; Parlato et al., 2017; Pavesi et al., 2017; Lee et al., 2018; Guo et al., 2019; Um et al., 2019; Yu et al., 2019; Ren et al., 2020; Ayuso et al., 2021), or through simple gravity-driven flow (Song et al., 2021), or perfusion with very low fluid flow rates, being far from physiological conditions. For instance, in a tumor-on-chip model, Aung et al. perfused T cells applying a fluid flow rate of 50 ul/hr (corresponding to 0,8 ul/min) (Aung et al., 2020); similarly, in an immune system-on-chip recently developed by Goyal et al., immune cells were cultured through a flow rate of 60 ul/hr (corresponding to 1 ul/min) (Goyal et al., 2022).

Instead, these devices could be further implemented with microfluidic motion, generating more reliable cell culture models with shear stresses and stimuli associated with fluid flows mimicking the dynamics of real tissues (e.g., interstitial flows, blood flows) as well as the biological and biochemical processes that physiologically rely upon dynamic flows (e.g., circulation of immune cells, drug kinetics). Here, we provide a fluid-dynamic technology for performing dynamic experiments with a 300 ul/min flow rate, corresponding to a physiological capillary blood flow velocity (Marrella et al., 2020; Marrella et al., 2021b), for mimicking the microcirculation of the TME. Specifically, NK cells were driven by the fluid flow motion below the tumor model, which was in turn cultured in a 3D matrix. NK cells and the tumor model were physically separated in two different compartments through a porous permeable membrane supporting the adoption of different culture media (e.g. serum percentage, selective growth factors), while NK cells were free to sense the chemo-attractive gradient exerted by tumor cells and to actively initiate a spontaneous “extravasation” process toward the tumor cells themselves.

Importantly, NK cells were able to migrate upward, against the gravity and viscous forces exerted by the fluid flow, demonstrating that their migration is specifically mediated by soluble factors released by tumor cells, as little or no migration has been observed in the negative control group (w/o chemo attractants, w/o tumor cells). In particular, the gradient of chemoattractant molecules activates the inner filament network of the cells, leading to a cell chemotactic response (Yang et al., 2015). This force that drives the cells to move towards the chemoattractant source is also named protrusion force. In our experiments, the protrusion force generated by the presence of tumor cells led to a migration of immune cells that was significantly higher than that obtained with increased serum percentage in the medium, demonstrating for the first time a tumor-mediated migration of immune cells in a biologically relevant organ-on-chip platform.

Interestingly, NK cells and tumor cells were co-cultured in the dynamic setting in a standard 10:1 E:T ratio (Ayuso et al., 2019; Sargenti et al., 2020; Gopal et al., 2021; Morimoto et al., 2021). However, we observed that only around 2% of circulating NK cells were able to specifically extravasate in the upper compartment, in line with the low number of NK cells generally observed in tumor tissues (Balsamo et al., 2012; Castriconi et al., 2018; Melaiu et al., 2020); then, only this cellular subset had the potential to really interact with tumor cells, highlighting the limitations of the current static co-culture modalities, where often high E/T ratios are used simply adding NK cells into the well/circuit, leading to a possible overestimation of their efficacy in tumor control. This technical issue of the current approaches might lead to a deep impact in the preclinical evaluation of immunotherapies, in which the activation and recruitment of specific subsets of immune cells (for drug-based therapies) as well as of infused, often engineered cells (for cell-based therapies) represent the crucial first step for their efficacy. Furthermore, this may explain at least in part the high success rate of immunotherapies at the preclinical level, which is not mirrored by the same success once translated into the clinic.

The second step for activated immune cells to be effective against the tumor is their capability to infiltrate the tumor bulk, keeping an activation state without being affected by the adverse/immunosuppressive signals from the TME. This is, for instance, the main challenge of CAR-T (and -NK) cell therapy for solid tumors: indeed, despite being successful for hematological malignancies, such cell-based therapy has not found a successful application for solid tumors yet (Guerra et al., 2021; Kumari et al., 2021). In this context, our experiments show that, beside the importance of determining the extravasation rate as indicative of immune cell recruitment, it is mandatory to assess the immune cell infiltration within real three-dimensional matrix-based tumor cultures, where the chemo-physics and biomechanics (i.e. stiffness) of the matrix itself better resemble the immune-tumor cross talk occurring *in vivo*. Importantly, the over-miniaturization of the “classical” microfluidic devices, beside failing in recapitulating the biological and clinical features of TME, possibly leads to the underrepresentation of the tumor heterogeneity occurring into the clinic. Moreover, this also carries some technical limitations related to downstream biochemical assays and to small volumes/bubbles handling (Ayuso et al., 2021; Song et al., 2021): indeed, the use of very small cell numbers (e.g., 1,000 cells/spheroid (Ayuso et al., 2019), 2,500 cells/well (Gopal et al., 2021)) and/or very small volumes (e.g., 10–20 ul containing 10^5^-2 × 10^5^ cells (Ren et al., 2020)) are not always suitable for standard analytical methods such as immunofluorescence and flow cytometry analysis. Consequently, the user adaptation to a different cell culture technology, with a less comfortable handling with respect to the standard cultures and a narrowed range of analytical methods (i.e., often confined to cell imaging) make these microfluidic devices not easy to adopt in conventional laboratory practices. On the contrary, following the approach here described, 3D cultures were obtained with more cells (min. 3 × 10^4^ cells/matrix) co-cultured with 10-fold higher NK circulating cells. Furthermore, given the increased sample size (up to 5 mm), our experiments represent the proof-of-concept for the use of this platform with more complex 3D cultures (i.e., based on bioprinted scaffolds and/or seeded with different cell types) to better mimic the TME, as well as with patient biopsies. The latter could have a profound impact on personalized screening, for tailoring patients based on the better response or for assessing the efficacy of cell-based therapies.

We already demonstrated the reliability of alginate-based cultures for different tumors (Cavo et al., 2018; Marrella et al., 2019; Marrella et al., 2021b), and in particular for NB cultures, where the susceptibility to therapies and the tumor cells immune-phenotype were properly predicted and recapitulated (Marrella et al., 2019). Here, we demonstrated that the alginate matrix is suitable also for culturing NK cells, without affecting their viability or their phenotype. Moreover, when NK cells and NB cells were co-embedded in alginate matrix, we observed that NK cells retained their ability to interact with tumor cells and to kill them, providing evidence of their migratory/infiltrating behavior within the hydrogel. After co-culturing NK cells and the 3D tumor in dynamic conditions, we also assessed the cytotoxic effect of infiltrated NK cells: since we observed a significant increase of annexin-V exposure on tumor cells, we demonstrated that NK cells also retained their killing capacity within the alginate matrix.

Importantly, we analyzed NK cell phenotype in terms of CD16 expression, within the circulating environment (administered NK cells), the migrated and infiltrated fractions, after 24 h of dynamic co-culture: we observed a clear decrease in CD16-positive NK cells within the recruited populations (both migrated and infiltrated). CD16^+^ cells represent a subpopulation of NK cells displaying cytotoxic activity higher than that exerted by the CD16^−^population (Orange, 2008; Myers and Miller, 2021). Since the infiltration and phenotype of NK cells have been correlated with prognosis and response to immunotherapy in NB tumors (Melaiu et al., 2020; Szanto et al., 2021), it is crucial to dispose of a preclinical model that faithfully recapitulates the human pathology.

In this context, our NB/NK model represents a paradigm for the establishment of advanced *in vitro* models that can be efficiently employed for testing immunotherapies also in different tumors, eventually in a personalized perspective. Here, by using HTLA-230, a human cell line highly recapitulating the most aggressive NB disease (Castriconi et al., 2007a), we provided a humanized and immunocompetent platform bridging the gap between standard *in vitro* methods, advanced miniaturized organ-on-chips and animal models. With its further optimization by the addition of cells and molecules characterizing the TME, this model could be more successfully utilized for deciphering or consolidating the mechanisms supposed to drive the quality and the amount of human NK cell infiltration in tumors (Castriconi et al., 2013; Regis et al., 2017). In the present model this infiltration could depend on the activity of factors such as TGF-β, MIF, or VEGF shown to be highly secreted by HTLA-230 cells line (Castriconi et al., 2013). Finally, these mechanisms could be finely tuned in combined personalized strategies to potentiate the efficacy of immunotherapies.

## Materials and methods

### Cell cultures

The MYC-N amplified neuroblastoma (NB) cell line HTLA-230 was provided by Dr. E. Bogenmann (Children’s Hospital Los Angeles, CA) (Corrias et al., 1996) and cultured in RPMI-1640 medium supplemented with 10% heat-inactivated FCS (Biochrom, Berlin, Germany), 50 mg/ml streptomycin, 50 mg/ml penicillin (Sigma-Aldrich), and 2 mm glutamine (Euroclone). The cells were cultured in a humidified environment (95% air/5% CO_2_) at 37°C and were used to generate 3D tumor models.

Peripheral blood mononuclear cells (PBMCs) were obtained from blood of volunteer healthy donors by Ficoll-Hypaque gradients (Sigma Aldrich). NK cells were purified by using the NK-cell isolation kit II (Miltenyi Biotec) and were cultured on irradiated PBMCs in RPMI-1640 supplemented with 10% heat-inactivated FCS, 50 mg/ml streptomycin, 50 mg/ml penicillin (Sigma-Aldrich), 2 mm glutamine (Euroclone), 600 IU/ml rhIL-2 (Proleukin; Chiron, Emeryville, CA) and 0.5% v/v phytohemagglutinin (Gibco, Paisley, United Kingdom). After 10 passages, NK cells were checked for purity (>95%) analyzing classical NK cell markers (Castriconi et al., 2007b).

### Computational fluid-dynamic simulations

Fluid dynamics within Single-Flow MIVO^®^ device was investigated to predict 1) the fluid velocity profiles within the device and 2) the fluid flow-driven NK cells trajectories.

First, the analysis was performed by using the Single-Phase Laminar Fluid Flow model of Comsol Multiphysics 5.6 assuming 1) a laminar flow regime, 2) an incompressible Newtonian fluid (Vitale et al., 2020; Pulsoni et al., 2022). The equations to be solved are the Navier-Stokes ones for the conservation of momentum Eq. 1 and the continuity law for conservation of mass Eq. 1:

$$\{\begin{array}{c} \rho \left[\frac{\partial u_{f}}{\partial t}+u_{f}\cdot \left(\nabla u_{f}\right)\right]=-\nabla p+\mu \left(\nabla^{2}u_{f}\right)\\ \left(\nabla \cdot u_{f}\right)=0\; \end{array}$$ (1)

where $u_{f}$ is the fluid velocity and p the pressure across the circuit. The values of the density *ρ* (1,000 kg/m^3^) and the viscosity *μ* (10^–3^ Pa⋅s) was selected as water at room temperature (25°C). A flow rate of 0.3 ml/min was set as input according to the value impose experimentally to generate the fluid motion, whereas as output the atmospheric pressure was set as null, avoiding a backflow. A no-slip boundary condition was set. Finally, an iterative geometric multigrid (GMRES) algorithm was used to solve the equations.

Subsequently, the particle tracing module for fluid flow was added to the model to identify the position and velocity of the NK cells dispersed in the moving fluid as a function of time. The Newtonian model was used to estimate the behaviour of the particles in the fluid flow. Such model is based on the conservation of total momentum:

$$\frac{d\left(m_{p}v_{p}\right)}{dt}=F_{d}+F_{g}$$ (2)

where m_p_ is the mass of the particle, v_p_ its velocity, F_d_ and F_g_ the drag force and the gravity force, respectively. The drag force was calculated through the Stokes drag law:

$$F_{d}=6\pi \mu r_{p}\left(u_{f}-v_{p}\right)$$ (3)

where µ is viscosity defined above, r_p_ is the particle radius (assuming NK cells as spheres) equal to 0.006 mm (Dickinson et al., 2015) The gravity force was calculated as:

$$F_{g}=m_{p}g\;\frac{\left(\rho_{p}-\rho \right)}{\rho_{p}}$$ (4)

where g is the gravity acceleration, ρ_p_ the particle density equal to 1,080 kg/m^3^ (Zhao et al., 2015) and ρ the fluid density defined above. A rebound condition on the walls was set, meaning that the momentum of the particles that encounter the wall is preserved.

### 3D cultures

Three-dimensional NB models were generated as previously described (Marrella et al., 2019). Briefly, HTLA-230 cells were resuspended in DMEM (Euroclone) supplemented with 10% heat-inactivated FBS, 1% penicillin/streptomycin and 1% glutamine and mixed with a 1% alginate solution (1:1 V/V), to obtain a final 0.5% alginate concentration (w/V). This cell suspension was dropped into a 0.5 M CaCl_2_ gelling bath gel spheroids formation. The hydrogels were then washed with sterile distilled water and transferred in a 96-well plate, cultured in DMEM supplemented with 10% FBS, 1% penicillin/streptomycin and 1% glutamine, and 5 mm CaCl_2_, ensuring gel maintenance.

For the establishment of NK cell 3D cultures, after thawing NK cells were recovered for 72 h, and then cultured for additional 24 h in the standard 2D setting (96-well plate) or embedded within the alginate matrix. Specifically, the 3D culture was obtained resuspending NK cells in DMEM supplemented with 10% FBS, 1% penicillin/streptomycin, 1% glutamine and 600 UI/ml IL2, and mixed with a 1% alginate solution (1:1 V/V). The 3D hydrogels were obtained following the procedure described above. The cells were then analyzed by flow cytometry in terms of cell viability through staining with a Cell Viability Dye.

For NB/NK cell 3D co-cultures, HTLA-230 cells or NK cells were resuspended in DMEM supplemented with 10% FBS, 1% penicillin/streptomycin and 1% glutamine or DMEM supplemented with 10% FBS, 1% pen/strep, 1% glutamine and 600 UI/ml IL2, respectively, and then mixed at different E:T ratios (1:1; 1:10). The 3D hydrogels were obtained following the procedure described above and kept in culture over/night before analyzing the expression of the NK marker CD16 through flow cytometry.

### Dynamic culture

The dynamic co-cultures were performed by using the Single-Flow MIVO^®^ device, with the aim to recapitulate the complexity of a 3D, dynamic TME. NK cells were counted, eventually stained with CFSE Cell Proliferation Kit (ThermoFisher Scientific), resuspended in RPMI-1640 medium (Euroclone) supplemented with 10% FBS, 1% penicillin/streptomycin, 1% glutamine and 600 UI/ml IL2, and loaded within the MIVO^®^ chamber (1.5 ml/chamber), to get an effector: target (E: T) ratio of 10:1 with tumor cells. The circulation of NK cells was allowed by a pumping system, through the imposition of a fluid flow rate of 0.3 ml/min, simulating capillary flows, while 3D NB hydrogels were cultured with the maintenance medium physically separated through a permeable porous membrane.

For extravasation experiments, after 4 h dynamic co-culture, both circulating (within the capillary circuit) and extravasating (within the tumor niche) NK cells were harvested and counted by means of a hemocytometer. NK cells were also cultured when the MIVO chamber contained either DMEM w/o supplement (negative control) or an alginate gel w/o NB cells supplemented with 40% FBS as a chemoattractant factor (positive control).

### Immunofluorescence analysis of 3D cultures

For 3D dynamic or static NK/NB co-cultures, NK cells were used unlabeled or labeled with carboxyfluorescein succinimidyl ester (CFSE) (CellTrace CFSE Cell proliferation kit, Molecular Probes). Briefly, NK cells were washed three times with RPMI-1640 and resuspended at 1 × 10^7^/ml in the same medium. CFSE was added at the final concentration of 100uM and incubated for 10 min at 37°C in water bath. The reaction was stopped by adding complete culture medium and cells were washed twice before performing the experiments.

For NK cell or NB staining, 3D cultures, either from dynamic or static conditions, were washed with a 0.9% sodium chloride solution supplemented with 5 mm CaCl_2_ and fixed in 4% paraformaldehyde supplemented with 1 mm CaCl_2_. For experiments performed with unlabeled NK cells, after incubation with blocking solution (2% BSA, 5 mm CaCl_2_ in 0.9% sodium chloride solution), the hydrogels were stained for the NK-associated marker DNAM-1 (Castriconi et al., 2004) (F5, mouse anti-human, IgM) primary antibody for 2 h, followed by a goat anti-mouse IgM FITC-conjugated secondary antibody (Southern Biotech, Birmingham, AL). Cell nuclei were counterstained with DAPI. The hydrogels were mounted on a microscope slide, squeezed with a glass coverslip, and then observed under a fluorescence microscope (Nikon ECLIPSE Ts2-FL).

For experiments performed with CFSE-labelled NK cells, after incubation with blocking solution (2% BSA, 5 mm CaCl_2_ in 0.9% sodium chloride solution), the hydrogels were stained for the NB-specific marker GD2, by using a direct Alexa Fluor 647 mouse anti-human Disialoganglioside GD2 (IgG2a, BD Biosciences) antibody. Cell nuclei were counterstained with DAPI. The 3D hydrogels were layered on a 0.17 mm-thick microscope coverslip (optically clear borosilicate glass) and examined on the laser scanning confocal microscope SP2-AOBS (Leica Microsystems, Mannheim, Germany), using either a 20x/0.70 (Plan Apochromat) objective or a HCX PL APO ×40/0.75–1.25 oil immersion objective, on a DM IRE2 inverted microscope. Fluorescent dye excitation was performed using a 488 nm laser for CFSE excitation (emission detection range 500–560 nm), a 633 nm laser for Alexa 647 excitation (emission detection range 655–760 nm), a 405 nm diode laser for DAPI excitation (emission detection range 410–480). Image merging was performed with Leica proprietary software or ImageJ.

To provide a formal proof of the NK cell-mediated tumor killing in 3D cultures, NB and NK cells were cultured alone or in co-cultures overnight (E:T 1:1). After cell recovering, cells were stained with direct Alexa Fluor 647 mouse anti-human GD2 (IgG2a, BD Pharmigen) and direct APC-H7 mouse anti-human CD45 (IgG1, BD Pharmigen) antibodies to discriminate NB cells (GD2+CD45^−^) from NK cells (GD2-CD45^+^). Then, cells were stained with Fixable Viability Stain 510 (BD Horizon) following manufacturer’s procedures and analyzed by flow cytometry (FACSVerse flow cytometer-BD).

### Flow cytometry analysis

To assess NK cell viability, cells were recovered from the 2D standard (96-well plate) culture or from the 3D culture. The latter was achieved by hydrogel dissolution, through incubation in an alginate solubilizing solution (0.15 M NaCl, 100 mm trisodium citrate dihydrate), for 10 min in a 37°C water bath. Cells were then washed with PBS w/o Ca^2+^ and Mg^2+^ and stained with a PE-TexasRed Fixable Cell Viability dye (ThermoFisher Scientific), following manufacturer’s protocol.

To assess NK cell infiltration within the 3D tumor culture, as well as their phenotype, the cells were recovered from the alginate scaffold as described above. Cells were also recovered from the medium surrounding the hydrogels within the transwell inserts (to analyze extravasated NK cells) and from the circulating compartment (to analyze circulating NK cells). The cells were then washed with PBS w/o Ca^2+^ and Mg^2+^ and incubated with the staining solutions, for 30 min: the cells were stained with an anti-human CD45-APC and an anti-human CD16-PacificBlue antibody (ThermoFisher Scientific). For the analysis of tumor cell death after dynamic co-culture, the cells were recovered from the alginate scaffold and subsequently stained with an anti-human CD45-APC antibody and an Annexin V-FITC Apoptosis Detection Kit (eBioscience).

After incubation with the staining solution, the cells were washed and run through a NovoCyte3000 Flow Cytometer System. Data were analyzed with the NovoExpress software (Agilent Technologies).

### Statistical analysis

Statistical analysis was performed with the GraphPad Software GraphPad Prism5. Differences between groups were assessed by *t* test, where applicable, or by one‐way analysis of variance (ANOVA. A *p* value < 0.05 was considered statistically significant), or by Mann-Whitney test (*p* value < 0.05 was considered statistically significant). At least three independent experiments have been performed with a minimum of three technical replicates.

## Data Availability

The original contributions presented in the study are included in the article/Supplementary Material, further inquiries can be directed to the corresponding author.
