# Correction: A multi-organ-on-chip to recapitulate the infiltration and the cytotoxic activity of circulating NK cells in 3D matrix-based tumor model

**DOI:** 10.3389/fbioe.2025.1737950

**Published:** 2026-01-22

**Authors:** Monica Marzagalli, Giorgia Pelizzoni, Arianna Fedi, Chiara Vitale, Fabrizio Fontana, Silvia Bruno, Alessandro Poggi, Alessandra Dondero, Maurizio Aiello, Roberta Castriconi, Cristina Bottino, Silvia Scaglione

**Affiliations:** 1 React4life S.r.l, Genoa, Italy; 2 Department of Biotechnology and Bioscience, University of Milano-Bicocca, Piazza Della Scienza, Milan, Italy; 3 National Research Council, CNR-IEIIT, Genoa, Italy; 4 Department of Experimental Medicine (DIMES), University of Genoa, Genoa, Italy; 5 Department of Pharmacological and Biomolecular Sciences (DiSFeB), University of Milan, Milan, Italy; 6 Molecular Oncology and Angiogenesis Unit, IRCCS Ospedale Policlinico San Martino, Genoa, Italy; 7 IRCCS Istituto Giannina Gaslini, Genoa, Italy

**Keywords:** immune-organ-on-chip, 3D human tumor model, natural killer cells, neuroblastoma, cells migration, cells infiltration

A correction has been made to the section Results, Dynamic culture and assessment of natural killer cell “extravasation”, Paragraph 3:

The authors described that they used 40% FBS as a chemoattractant: “To this purpose, additional experimental groups included 1) a positive control of cell migration (“empty” 3D alginate scaffolds, without tumor cells, in culture medium enriched with 40% FBS as a chemoattractant), and 2) a negative control (without alginate scaffolds, with culture medium without any chemoattracting supplement).”

However, the authors used 30%. The corrected paragraph is:

*“To this purpose, additional experimental groups included 1) a positive control of cell migration (“empty” 3D alginate scaffolds, without tumor cells, in culture medium enriched with 30% FBS as a chemoattractant), and 2) a negative control (without alginate scaffolds, with culture medium without any* chemoattracting *supplement).”*

A correction has been made to the section Results, Dynamic culture and assessment of natural killer cell “extravasation”, Paragraph 3:

“the stimulation does not report cells “extravasation” events from the bottom chamber towards the tumor, because of the fluid motion alone, as shown in Figure 6C.”

“Figure 6C” should be “Figure 6A.” The corrected paragraph is:

“the stimulation does not report cells “extravasation” events from the bottom chamber towards the tumor, because of the fluid motion alone, as shown in Figure 6A”.

A correction has been made to the section Results, Natural killer cell infiltration, Paragraph 1:

“Figure 6D” is mentioned incorrectly and should be changed to “Figure 6C.” The corrected paragraph is:

“After co-culturing the cells for 4 h under microcirculation, the 3D alginate cultures were recovered, fixed with 4% PFA, NK cells stained for the DNAM-1 marker (Castriconi et al., 2004), and observed under a fluorescence microscopy. As shown in Figure 6C, DNAM-1+ cells were mostly located along the border of the gels. This is indicative of the ability of NK cells not only to specifically migrate toward the tumor culture, but also to infiltrate the alginate matrix.”

The original article has been updated.

